# Roles of circular RNAs in the pathogenesis and treatment of pancreatic cancer

**DOI:** 10.3389/fcell.2022.1023332

**Published:** 2022-11-17

**Authors:** Takahiro Seimiya, Motoyuki Otsuka, Mitsuhiro Fujishiro

**Affiliations:** Department of Gastroenterology, Graduate School of Medicine, The University of Tokyo, Tokyo, Japan

**Keywords:** circular RNA, circRNA, pancreatic cancer, biomarker, treatment

## Abstract

Circular RNAs are single-stranded RNAs with a covalently closed structure formed by the process of back-splicing. Aberrant expression of circular RNAs contributes to the pathogenesis of a wide range of cancers. Pancreatic cancer is one of the most lethal cancers due to diagnostic difficulties and limited therapeutic options. Circular RNAs are emerging as novel diagnostic biomarkers and therapeutic targets for pancreatic cancer. Moreover, recent advances in the therapeutic application of engineered circular RNAs have provided a promising approach to overcoming pancreatic cancer. This review discusses the roles of circular RNAs in the pathogenesis of pancreatic cancer and in potential treatment applications and their usefulness as diagnostic biomarkers.

## 1 Introduction

Pancreatic cancer is the seventh leading cause of cancer-related death worldwide, responsible for approximately 470 000 deaths per year (Sung et al., 2021). The number of deaths related to pancreatic cancer is increasing, and the prognosis remains poor, with a 5-year survival rate of only approximately 11% (Siegel et al., 2022). This poor prognosis is because most patients are diagnosed at advanced stages, and there is a lack of effective treatment options. In fact, the 5-year survival rates of patients with stage 0 and stage IV pancreatic cancer are 85.8% and 2.7%, respectively (Egawa et al., 2012). To improve the prognosis of pancreatic cancer, it is necessary to elucidate the molecular mechanisms underlying the pathogenesis of pancreatic cancer, to enable the development of methods for early diagnosis and effective treatment.

Recent progress in research on circular RNAs (circRNAs), which are single-stranded RNAs with a covalently closed structure, has shown that many circRNAs are dysregulated and involved in the pathogenesis of cancers (Vo et al., 2019). The presence of circRNAs was first reported in pathogens, such as plant viroids (Sanger et al., 1976) and hepatitis delta virus (Kos et al., 1986), followed by the discovery in the 1990s of circRNAs among human transcripts (Nigro et al., 1991; Cocquerelle et al., 1992, 1993; Capel et al., 1993). Over the last decade, advances in RNA sequencing technology and analytical methods have begun to reveal the overall picture of circRNAs in the human transcriptome. To date, more than 180 000 circRNAs have been identified, and their expression patterns have been shown to vary according to tissue or cell type and disease state (Jeck et al., 2013; Memczak et al., 2013; Salzman et al., 2013; Rybak-Wolf et al., 2014; Gao et al., 2015; Dong et al., 2018).

With clarification of the diversity of circRNAs in the human transcriptome, their biological functions are also being elucidated. This review discusses the roles of circRNAs in the pathogenesis of pancreatic cancer and potential therapeutic applications and their utility as diagnostic markers for pancreatic cancer.

## 2 Roles of circRNAs in the pathogenesis of pancreatic cancer

**FIGURE 1:**
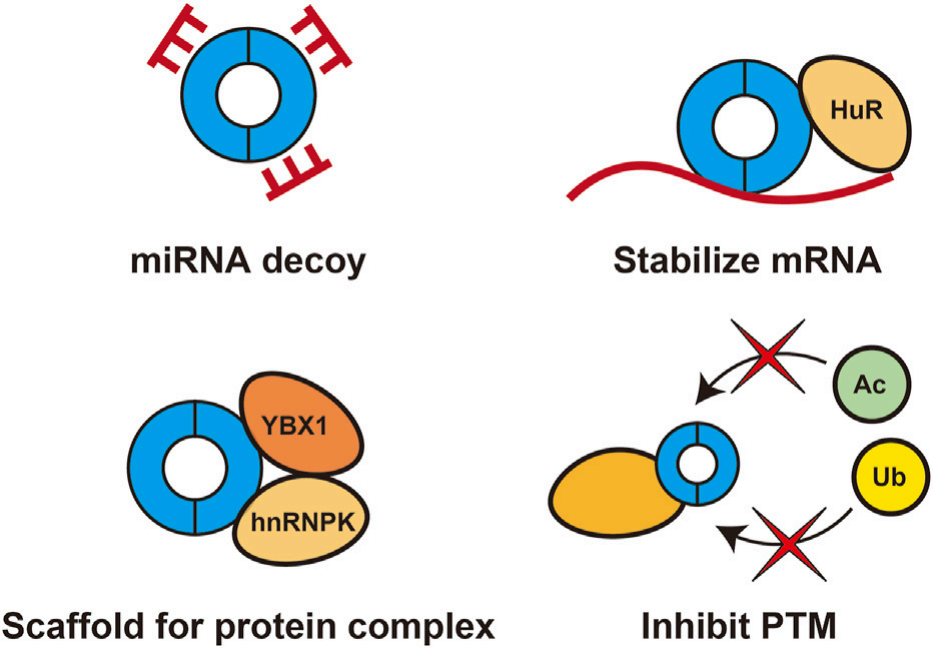
Roles of pathogenic circRNAs in pancreatic cancerPathogenic circRNAs in pancreatic cancer function as miRNA decoys and interact with proteins to stabilize mRNA, inhibit post-translational modifications (PTMs), and provide scaffolds for protein complex formation.

Although circRNAs have identical exon sequences as those of their linear cognate mRNAs, they have distinct cellular functions. circRNAs can interact with DNA, RNA, and proteins to modulate transcription, act as decoys for miRNAs and proteins, enhance mRNA stability, provide scaffolds for protein complex formation, and serve as templates for translation. In pancreatic cancer, many circRNAs are dysregulated and play important roles in various aspects of cancer progression. Most pathogenic circRNAs function as miRNA decoys, and several circRNAs have been reported to interact with proteins to stabilize mRNA, inhibit post-translational modifications, and provide scaffolds for formation of protein complexes (Figure 1). This chapter focuses on representative circRNAs involved in the pathogenesis of pancreatic cancer for which the detailed mechanisms have been elucidated, and those that could not be introduced in the text are summarized in Table 1.

**TABLE 1:** Pathogenic circRNAs in pancreatic cancer.

circRNA	Mechanism	Target	Biological function	Reference
circRNA_100782	miRNA decoy	miR-124/IL6R	Promotes proliferation	Chen et al. (2017)
circIARS	miRNA decoy	miR-122/RhoA	Increases endothelial permeability	Li et al. (2018a)
circPDE8A	miRNA decoy	miR-338/MACC1	Promotes invasion	Li et al. (2018b)
circZMYM2	miRNA decoy	miR-335-5p/JMJD2C	Promotes proliferation and invasion; inhibits apoptosis	An et al. (2018)
circADAM9	miRNA decoy	miR-217/PRSS3	Promotes proliferation and invasion	Xing et al. (2019)
circASH2L	miRNA decoy	miR-34a/Notch1	Promotes proliferation, invasion, and angiogenesis	Chen et al. (2019)
circLDLRAD3	miRNA decoy	miR-137-3p/PTN	Promotes proliferation, invasion, and migration	Yao et al. (2019)
hsa_circRNA_0007334	miRNA decoy	miR-144-3p and miR-577/MMP7 and COL1A1	Promotes migration	Yang et al. (2019)
circ_0000977	miRNA decoy	miR-153/HIF1A	Helps HIF1A-mediated immune escape	Ou et al. (2019)
ciRS-7 (CDR1as)	miRNA decoy	miR-7	Promotes proliferation and invasion	Liu et al. (2019)
circ_0030235	miRNA decoy	miR-1253 and miR-1294	Promotes proliferation and invasion; inhibits apoptosis	Xu et al. (2019)
circRHOT1	miRNA decoy	miR-26b, miR-125a, miR-330 and miR-382	Promotes proliferation, invasion, and migration	Qu et al. (2019)
circ_0007534	miRNA decoy	miR-625 and miR-892b	Promotes proliferation, invasion, and migration	Hao et al. (2019)
circRNA_000864	miRNA decoy	miR-361-3p/BTG2	Suppresses migration and invasion	Huang et al. (2020)
circ_0075829	miRNA decoy	miR-1287-5p/LAMTOR3	Promotes proliferation, invasion, and migration	Zhang et al. (2020b)
chr7:154954255-154998784+	miRNA decoy	miR-4459/KIAA0513	Promotes proliferation	Shao et al. (2020)
circ_0013912	miRNA decoy	miR-7-5p	Promotes proliferation and metastasis	Guo et al. (2020a)
hsa_circRNA_001587	miRNA decoy	miR-223/SLC4A4	Inhibits migration, invasion, and angiogenesis	Zhang et al. (2020a)
circRHOT1	miRNA decoy	miR-125a-3p/E2F3	Promotes proliferation and invasion, inhibit apoptosis	Ling et al. (2020)
circBFAR	miRNA decoy	miR-34b-5p/MET/Akt	Promotes proliferation and migration	Guo et al. (2020b)
circNFIB1	miRNA decoy	miR-486-5p/PIK3R1/VEGF-C	Inhibits lymphangiogenesis and lymphatic metastasis	Kong et al. (2020)
circFOXK2	miRNA decoy and protein interaction	miR-942/ANK1, GDNF, PAX6 and scaffold for YBX1 and hnRNPK	Promotes proliferation, invasion, and migration	Wong et al. (2020)
hsa-circ-001653	miRNA decoy	miR-377/HOXC6	Promotes cell-cycle progression, *in vitro* angiogenesis, and invasion	Shi et al. (2020)
circHIPK3	miRNA decoy	miR-330-5p/RASSF1	Promotes gemcitabine resistance	Liu et al. (2020)
circSEC24A	miRNA decoy	miR-606/TGFBR2	Promotes proliferation, invasion, and migration	Chen et al. (2021)
circRNF13	miRNA decoy	miR-139-5p/IGF1R	Promotes proliferation, invasion, and migration	Liu et al. (2021c)
circ_0092367	miRNA decoy	miR-1206/ESRP1	Inhibits EMT and gemcitabine resistance	Yu et al. (2021)
circ_0013587	miRNA decoy	miR-1227/E-Cadherin	Inhibits resistance to erlotinib	Xu et al. (2021a)
hsa_circ_0006117	miRNA decoy	miR-96-5p/KRAS/MAPK	Promotes proliferation, invasion, and migration	Liu et al. (2021a)
circPTPN22	Protein interaction	Inhibit STAT3-SIRT1 interaction	Attenuates immune microenvironment	He et al. (2021)
circEYA3	miRNA decoy	miR-1294/c-Myc	Increases energy production via ATP synthesis	Rong et al. (2021a)
circRHOBTB3	miRNA decoy	miR-600/NACC1/Akt/mTOR	Promotes autophagy and proliferation	Yang et al. (2021)
circ_0099999	miRNA decoy	miR-330-5p/FSCN1	Promotes proliferation, invasion,, migration, and glycolysis	Wang et al. (2021)
circZNF91	miRNA decoy	miR-23b-3p/SIRT1	Exosomal transmission to induce chemoresistance	Zeng et al. (2021)
circCCT3	miRNA decoy	miR-613/VEGFA/VEGFR2	Promotes proliferation and invasion, inhibit apoptosis	Hou et al. (2021)
circ-0005105	miRNA decoy	miR-20a-3p/COL11A1	Promotes EMT	Ma et al. (2021)
circSLIT2	miRNA decoy	miR-510-5p/c-Myc/LDHA	Promotes glycolysis and proliferation	Guan et al. (2021)
circ_0066147	miRNA decoy	miR-326/E2F2	Promotes proliferation and invasion, inhibit apoptosis	Zhang and Zhang, (2021)
circ_0001666	miRNA decoy	miR-1251/SOX4	Promotes EMT and invasion	Zhang et al. (2021a)
circPCDH10	miRNA decoy	miR-338-3p/hTERT	Promotes proliferation and invasion	Zhang et al. (2021b)
circ_0030167	miRNA decoy	miR-338-5p/Wif1/Wnt8/β-catenin	Exosomal transmission to inhibit proliferation, invasion, and migration	Yao et al. (2021)
circ_03955	miRNA decoy	miR-3662/HIF-1α	Inhibits apoptosis and promotes Warburg effect	Liu and Xu, (2021)
circ_0092314	miRNA decoy	miR-671/S100P	Promotes EMT	Shen et al. (2021b)
circ-MBOAT2	miRNA decoy	miR-433-3p/GOT1	Promotes proliferation and invasion; inhibits apoptosis	Zhou et al. (2021)
circNEIL3	miRNA decoy	miR-432-5p/ADAR1	Promotes EMT	Shen et al. (2021a)
CDR1as	miRNA decoy	miR-432-5p/E2F3	Promotes proliferation and invasion	Xiong et al. (2021)
circRNA_000684	miRNA decoy	miR-145/KLF5	Promotes proliferation and invasion, and migration	Liu et al. (2021b)
hsa_circ_0071036	miRNA decoy	miR-489	Promotes proliferation and invasion; inhibits apoptosis	Han et al. (2021)
circRNA_102049	miRNA decoy	miR-455-3p/CD80	Promotes proliferation and invasion; inhibits apoptosis	Zhu et al. (2021)
circEIF6	miRNA decoy	miR-557/SLC7A11/PI3K/AKT	Promotes proliferation and invasion; inhibits apoptosis	Zhang et al. (2021c)
hsa_circ_0050102	miRNA decoy	miR-1182/NPSR1	Promotes proliferation and invasion; inhibits apoptosis	Hua et al. (2021)
circSFMBT1	miRNA decoy	miR-330-5p/PAK1	Promotes proliferation and invasion	Xu et al. (2021c)
circ-MTHFD1L	miRNA decoy	miR-615-3p/RPN6	Promotes gemcitabine resistance	Chen et al. (2022b)
circ_0047744	miRNA decoy	miR-21/SOCS5	Suppresses metastasis	Xie et al. (2022)
circATG7	miRNA decoy and protein interaction	miR-766-5p/ATG7 and HuR interaction	Promotes autophagy and proliferation	He et al. (2022)
circCUL2	miRNA decoy	miR-203a-5p/MyD88/NFkB/IL6	Induces inflammatory cancer-associated fibroblast	Zheng et al. (2022)
circANAPC7	miRNA decoy	miR-373/PHLPP2/AKT/TGFb	Promotes proliferation and muscle wasting	Shi et al. (2022)
circ_0072008	miRNA decoy	miR-545-3p/SLC7A11	Promotes proliferation, invasion, and glycolysis	Sun et al. (2022)
hsa_circ_0050102	miRNA decoy	miR-218-5p/PPME1	Promotes proliferation, invasion, and angiogenesis	Feng et al. (2022)
circFARP1	miRNA decoy and protein interaction	miR-660-3p/LIF and CAV1 interaction	Promotes gemcitabine resistance	Hu et al. (2022)
circRTN4	miRNA decoy and protein interaction	miR-497-5p/HOTTIP/HOXA13 and RAB11FIP interaction	Promotes proliferation and invasion; inhibits apoptosis	Wong et al. (2022)
circUHRF1	miRNA decoy	miR-1306-5p/ARL4C	Promotes proliferation, migration, and EMT, inhibits apoptosis	Liu et al. (2022)
hsa_circ_0074298	miRNA decoy	miR-519d/SMOC2	Promotes proliferation and gemcitabine resistance	Hong et al. (2022a)

### 2.1 Decoys for miRNAs

As first demonstrated with CDR1as, some circRNAs function as decoys that bind to miRNAs and prevent them from binding to their target mRNAs (Hansen et al., 2013; Memczak et al., 2013). Hansen et al. also demonstrated that CDR1as reduced miR-7 activity, whereas the cognate linear RNA of CDR1as had little effect on miR-7 activity, suggesting that circRNAs have unique biological functions that differ from linear RNAs despite having the same sequence. In pancreatic cancer, many pathogenic circRNAs have been reported to function as miRNA decoys, leading to cell proliferation, inhibition of apoptosis, metastasis, chemotherapy resistance, and metabolic reprogramming. For example, circBFAR was identified as an upregulated circRNA in pancreatic cancer, and its overexpression was shown to be correlated with poor prognosis (Guo X. et al., 2020). Mechanistically, circBFAR binds to miR-34b-5p and decreases MET expression, thereby activating the MET/PI3K/Akt signaling pathway and promoting the progression of pancreatic cancer cells. In another example, circSLIT2 was shown to upregulate c-Myc expression by binding to miR-510-5p and to facilitate aerobic glycolysis in pancreatic cancer cells (Guan et al., 2021). In addition, many other circRNAs have been reported to contribute to pancreatic cancer progression in a cell-autonomous manner by disrupting miRNA function.

**FIGURE 2:**
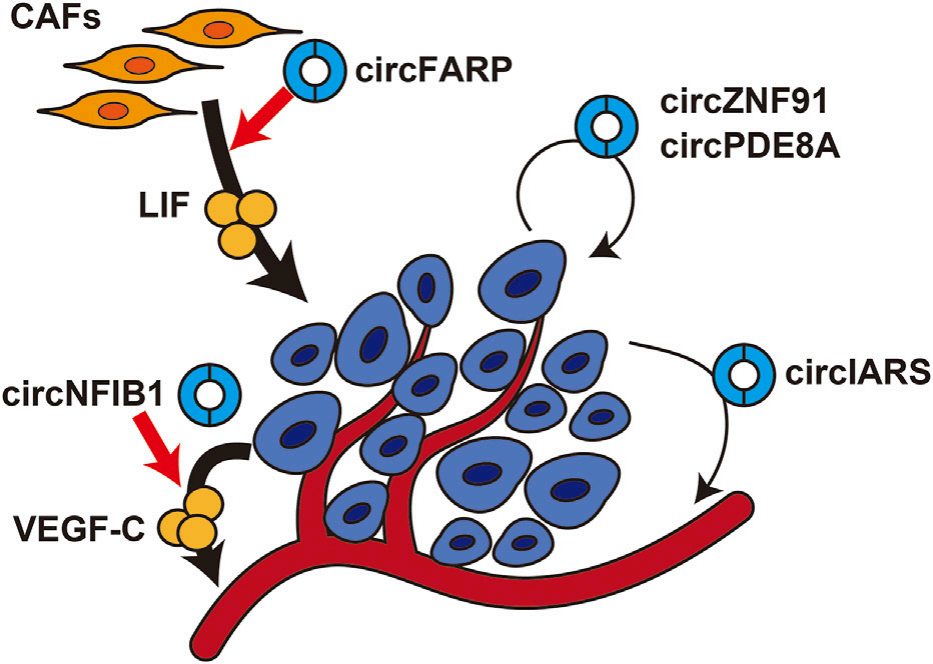
Effects of circRNAs on the tumor microenvironment in pancreatic cancercircFARP enhances LIF expression in cancer-associated fibroblasts. circNFIB1 enhances VEGF-C expression in pancreatic cancer cells. circZNF91 and circPDE8A are transmitted from pancreatic cancer cells to nearby cancer cells. circIARS is transmitted from pancreatic cancer cells to endothelial cells.

The circRNAs that are dysregulated in cancer cells not only increase their malignant potential in a cell-autonomous manner but also contribute to the progression of pancreatic cancer in a non-cell-autonomous manner by altering the tumor microenvironment (Figure 2). circNFIB1 was shown to be downregulated and to be negatively associated with lymph node metastasis in pancreatic cancer patients (Guan et al., 2021). circNFIB1 functions as a decoy for miR-486-5p and antagonizes the miR-486-5p-mediated suppression of PIK3R1 expression. As PIK3R1 acts as an inhibitor of the PI3K/Akt signaling pathway, circNFIB1 inhibits the PI3K/Akt pathway and downregulates its downstream target, VEGF-C, resulting in suppression of lymphangiogenesis and lymph node metastasis.

Cancer-associated fibroblasts (CAFs) are the predominant cell type in the stroma of pancreatic cancer and play an important role in chemotherapy resistance. circFARP was identified as a circRNA specifically upregulated in gemcitabine-resistant CAFs, and high circFARP expression was shown to be correlated with poor progression-free survival (Guan et al., 2021). When pancreatic cancer cells were cultured in conditioned medium derived from circFARP-overexpressing CAFs, they acquired gemcitabine resistance. Mechanistically, circFARP enhances leukemia inhibitory factor (LIF) expression by decoying miR-660-3p, and LIF secreted from CAFs induces gemcitabine resistance in pancreatic cancer cells by activating the LIF/STAT3 pathway. Interestingly, circFARP inhibits CAV1 degradation by interacting directly with CAV1, which promotes caveola-mediated exocytosis of LIF. Thus, circFARP functions as both a miRNA decoy and protein decoy.

Recent studies have shown that circRNAs are enriched in exosomes and may mediate intercellular communication between cancer cells and the tumor microenvironment to promote cancer progression (Seimiya et al., 2020). For example, circPDE8A was identified as a circRNA enriched in exosomes secreted from liver metastatic pancreatic cancer cells (Li Z. et al., 2018). circPDE8A functions as a decoy for miR-338 to upregulate MACC1 and stimulates invasive growth *via* the MACC/MET/ERK or AKT pathway. Exosomes derived from circPDE8A-overexpressing cancer cells activate the MACC/MET/ERK or AKT pathway and promote epithelial–mesenchymal transition. In clinical practice, circPDE8A was also detected in exosomes from the plasma of pancreatic cancer patients, and high exosomal circPDE8A expression was shown to be correlated with TNM stage and poor prognosis. circIARS was also enriched in exosomes from liver metastatic pancreatic cancer cells (Li J. et al., 2018). Exosomal circIARS decoyed miR-122 to increase RhoA expression, which in turn increased the permeability of human umbilical vein endothelial cells and facilitated invasion of pancreatic cancer cells. Another study showed that hypoxia-induced exosomal circZNF91 promoted chemoresistance in normoxic pancreatic cancer (Zeng et al., 2021). circZNF91 was upregulated in exosomes derived from pancreatic cancer cells under hypoxic conditions compared with normoxic conditions. When hypoxia-induced exosomal circZNF91 was transmitted to normoxic tumor cells, circZNF91 bound to miR-23b-3p and upregulated SIRT1 expression. Consequently, SIRT1 stabilized HIF-1α protein, which enhanced glycolysis and promoted chemoresistance in recipient cells. These results suggest that exosomal circRNAs may function as decoys for miRNAs in recipient cells and play important roles in cancer progression by mediating signal transmission between pancreatic cancer cells and the tumor microenvironment.

Although there is accumulating evidence that circRNAs are involved in the progression of pancreatic cancer, caution is required when considering whether a given circRNA has a measurable effect on cellular functions by acting as an miRNA decoy. The most well-studied circRNA with miRNA decoy function is CDR1as, which has over 70 binding sites for miR-7 (Hansen et al., 2013). Another well-known circRNA has 16 miR-138 target sequences (Hansen et al., 2013). Compared with these well-known circRNAs, most circRNAs have few miRNA-binding sites and low expression levels (Guo et al., 2014). Therefore, when investigating the potential of a circRNA of interest to act as a miRNA decoy and contribute to the pathogenesis of pancreatic cancer, careful consideration of whether the expression of that circRNA and the number of miRNA-binding sites are sufficient to achieve a measurable effect is needed.

### 2.2 Inhibiting post-translational modifications

Some circRNAs interact directly with proteins and stabilize them by inhibiting ubiquitin–proteasomal degradation. For example, circFARP1, which is upregulated in CAFs, interacts with CAV1 and blocks the interaction between CAV1 and the E3 ubiquitin ligase ZNRF1 to inhibit CAV1 degradation (Hu et al., 2022). As CAV1 mediates caveola-mediated exocytosis, CAV1 stabilization enhances LIF exocytosis from CAFs and activates the JAK–STAT signaling pathway in pancreatic cancer cells. As another example, circRTN4 interacts with RAB11FIP, which plays an important role in epithelial–mesenchymal transition, and protects it from ubiquitin–proteasomal degradation (Wong et al., 2022). Prediction of the structure of the circRTN4–RAB11FIP1 complex revealed that circRTN4 blocked the Lys578 ubiquitination site of RAB11FIP1. These results suggest that circRNAs bind to proteins and enhance their stability by inhibiting ubiquitin–proteasomal degradation.

It has been suggested that circPTPN22 inhibits protein–protein interactions and promotes STAT3 acetylation (He et al., 2021). circPTPN22 is upregulated in pancreatic cancer, and it interacts directly with STAT3 to block the interaction between STAT3 and SIRT1. In a xenograft model, knockdown of circPTPN22 enhanced intratumoral T cell infiltration and inhibited tumor growth. These results suggest that circPTPN22 inhibits STAT3 deacetylation by SIRT1 and promotes immune evasion of pancreatic cancer.

### 2.3 Scaffolds for protein complexes

circRNAs also serve as scaffolds for mRNA–protein complex formation to enhance expression of the mRNA. circFOXK2 is highly expressed in pancreatic cancer and promotes cell proliferation, migration, and invasion (Wong et al., 2020). Pulldown of circRNAs followed by mass spectrometry revealed that circFOKX2 interacts with YBX1 and hnRNPK. The YBX1–hnRNPK complex is reportedly involved in cancer progression. To determine the direct targets of the YBX1–hnRNPK complex, a circFOKX2 RNA pulldown assay and RNA immunoprecipitation assay of YBX1 and hnRNPK were performed, and NUF2 and PDXK mRNA were identified as direct targets. Knockdown of circFOXK2 downregulated the expression of NUF2 and PDXK mRNA and inhibited the interactions of YBX1 and hnRNPK with NUF2 and PDXK mRNA. These results suggest that circFOXK2 provides a scaffold for the YBX1–hnRNPK complex to promote expression of NUF2 and PDXK. Another study showed that circATG7 interacted with HuR and increased the mRNA level of ATG7 (He et al., 2022). circATG7 exists in both the cytoplasm and nucleus; cytoplasmic circATG7 acts as a decoy for miR-766-5p to decrease the expression of its target gene, ATG7, while nuclear circATG7 interacts with HuR and increases the stability of ATG7 mRNA in a HuR-dependent manner.

Taken together, these observations suggest that pathogenic circRNAs in pancreatic cancers function as miRNA decoys and/or interact with proteins. However, given that the exact mechanisms of action of many circRNAs remain unknown, that circRNAs acting as templates for protein translation have not been identified in pancreatic cancer, and that a single circRNA can have multiple functions, the impact of circRNAs on the progression of pancreatic cancer may be much greater than known at present. Further studies are needed to elucidate the overall impact of circRNAs on the pathogenesis of pancreatic cancer.

## 3 circRNAs as diagnostic biomarkers

### 3.1 Pancreatic cancer

Although early diagnosis is important to improve the prognosis of pancreatic cancer, most pancreatic cancer cases are diagnosed at an advanced stage. One reason for this is that pancreatic cancer biomarkers are ineffective for early diagnosis. For example, serum pancreatic enzymes, such as amylase, are elevated in only 20–50% of patients with pancreatic cancer, and serum CA19-9, which is routinely used as a biomarker for pancreatic tumors, is elevated in 70–90% of cases (Sharma et al., 2011). However, the sensitivity of CA19-9 for detecting pancreatic cancer lesions ≤2 cm decreases to approximately 50% (Liu et al., 2012), which is insufficient for early diagnosis. Therefore, the development of novel diagnostic biomarkers is required.

circRNAs have a half-life of 18.8–23.7 h, which is significantly longer than that of their cognate mRNAs (4.0–7.4 h) (Enuka et al., 2016). In addition, circRNAs exhibit characteristic expression patterns in different types of cancer (Vo et al., 2019). Due to their remarkable stability and specificity, circRNAs are regarded as potential diagnostic biomarkers for cancers. To date, five studies have reported the utility of serum circRNAs as diagnostic biomarkers for pancreatic cancer (Table 2). That is, circLDLRAD3, circPDAC, hsa_circ_0013587, and circ_001569 were reported to be aberrantly expressed in the serum of pancreatic cancer patients (Yang et al., 2017; Xu K. et al., 2021; Shen X. et al., 2021; Seimiya et al., 2021), and another study suggested that the combination of hsa_circ_0006220 and hsa_circ_0001666 can improve diagnostic performance compared with each circRNA alone (Hong L. et al., 2022). These studies demonstrated that circRNAs have a sensitivity of 0.45–0.76 and specificity of 0.70–0.90 for diagnosis of pancreatic cancer.

**TABLE 2:** CircRNAs as diagnostic biomarkers for pancreatic cancer.

circRNA	Sensitivity	Specificity	Mechanism/Target/Biological function	Reference
circ-LDLRAD3	0.57	0.70	miRNA decoy/miR-137-3p/Promotes proliferation, invasion, and migration	Yang et al. (2017)
circPDAC	0.45	0.90	Unknown	Seimiya et al. (2021)
hsa_circ_0013587	0.76	0.76	miRNA decoy/miR-1227/Inhibits resistance to erlotinib	Xu et al. (2021b)
circ_001569	0.63	0.74	Unknown	Shen et al. (2021c)
hsa_circ_0006220 and hsa_circ_0001666	0.74	0.87	Unknown	Hong et al. (2022b)

### 3.2 Intraductal papillary mucinous neoplasm

It has been suggested that circRNAs may be useful for diagnosis of not only pancreatic cancer but also intraductal papillary mucinous neoplasm (IPMN), which is a precancerous lesion of pancreatic cancer (Seimiya et al., 2021). It was shown that circPDAC is highly expressed in pancreatic cancer, and that high circPDAC expression is associated with lymph node metastasis and cancer stage. This circRNA is enriched in exosomes and can be detected in the blood of patients with pancreatic cancer, suggesting that measurement of circPDAC levels in blood may be useful for the diagnosis of pancreatic cancer. Interestingly, circPDAC was also detected in the blood of patients with IPMN. As there are currently no effective biomarkers for diagnosis of IPMN, circPDAC may serve as a novel diagnostic biomarker for this disease. Recently, pancreatic surveillance of high-risk individuals with genetic and familial risk factors for pancreatic cancer was shown to lead to early diagnosis and better long-term survival (Dbouk et al., 2022). In the same way, pancreatic surveillance in patients with IPMN may also reduce pancreatic cancer mortality through early detection.

A number of issues remain to be resolved for the routine application of circRNAs as diagnostic biomarkers for pancreatic cancer or IPMN in real-world clinical practice. First, because of the relatively small number of cases in the studies discussed above, it will be necessary to evaluate the diagnostic performance in larger cohorts including patients with early-stage pancreatic cancer. In addition, PCR is commonly used to measure the expression levels of circRNAs in blood, but PCR is labor-intensive and requires multiple steps, including RNA extraction from blood, reverse transcription, and cDNA amplification. Therefore, it is necessary to develop a simpler and more sensitive measurement method. Future research will resolve these issues and provide hope for early diagnosis of pancreatic cancer.

## 4 Therapeutic applications of circular RNAs

### 4.1 circRNAs as therapeutic molecules

As circRNAs have unique biological functions and molecular characteristics, they have potential applications as new nucleic acid therapeutic agents. One possible application is to use circRNAs as stable molecules for RNA interference therapeutics. For example, engineered circRNA molecules encoding miR-34a-3p and -5p sequences, called db34a RNA, significantly inhibited angiogenesis of pancreatic cancer cells (Gnanamony et al., 2021). This is the only study to date that has examined the potential application of circRNAs as therapeutic molecules in pancreatic cancer, but a variety of applications are being investigated in other diseases, which have been reviewed elsewhere (Liu and Chen, 2022). Briefly, based on the stability and biological functions of circRNAs, various applications of these RNAs are being investigated, including as decoys for miRNAs and proteins, stable antisense RNAs, boosters of innate immune responses, inhibitors of innate immune responses, and templates for protein translation. These various circRNA-based therapies may also be applicable in pancreatic cancer.

### 4.2 Targeting pathogenic circRNAs

As the roles of circRNAs in the pathogenesis of pancreatic cancer become clearer, new therapeutic approaches targeting pathogenic circRNAs are also expected. For example, circRTN4 is highly expressed in pancreatic cancer and is correlated with liver metastasis (Wong et al., 2022). Knockdown of circRTN4 in pancreatic cancer cell lines inhibits their proliferation, migration, and invasion. Furthermore, circRTN4 knockdown significantly suppressed tumor growth and metastasis to the liver in a mouse xenograft model. In addition, many pathogenic circRNAs have also been reported to be potential therapeutic targets.

Although many circRNAs are expected to be therapeutic targets, several challenges remain for clinical application of circRNA-targeted therapies. Antisense oligonucleotides, siRNAs, shRNAs, and CRISPR–Cas systems can be used to target RNAs. Clinical application of CRISPR–Cas systems is being intensively evaluated, and several oligonucleotide drugs have been approved by the FDA and are clinically available (Roberts et al., 2020; Modell et al., 2022). To apply these molecules to circRNA-targeted therapies, they should specifically recognize back-splice junctions. However, because circRNAs have identical sequences to those of their cognate linear mRNAs, in cases where the back-splice junction has a similar nucleotide sequence to that of a canonical splicing junction, it may not be possible to engineer therapeutic molecules that specifically recognize circRNAs. Even with proper design, care is required regarding off-target effects, especially those on cognate linear mRNAs. A number of processes are involved in the regulation of circRNA expression, including transcription, splicing, nuclear export, and degradation (Chen, 2020). Elucidating these molecular mechanisms may provide new therapeutic targets for regulating circRNA expression levels.

## 5 Concluding remarks

With the development of RNA sequencing methods for circRNAs, a great deal of progress in the research of these molecules has been made over the past decade. Accordingly, some circRNAs have been shown to play important roles in a variety of pathological conditions, including pancreatic cancer (Rong et al., 2021b; Chen Q. et al., 2022). However, the functional significance of many circRNAs is largely unknown. They may be meaningless byproducts generated during the splicing process, but they may have important biological functions that remain to be elucidated. It is hoped that the currently unknown pathological roles of circRNAs will be further elucidated, and new therapeutic strategies for pancreatic cancer using circRNAs will be developed in the future.
